# Loss of TC-PTP in keratinocytes leads to increased UVB-induced autophagy

**DOI:** 10.1038/s41420-025-02353-8

**Published:** 2025-02-28

**Authors:** Obed Asare, Lindsey Shim, Cheol-Jung Lee, Jose Delgado, Natasha Quailes, Klarissa Zavala, Junsoo Park, Bilal Bin Hafeez, Yong-Yeon Cho, Subhash C. Chauhan, Dae Joon Kim

**Affiliations:** 1 Department of Medicine and Oncology, School of Medicine, University of Texas Rio Grande Valley, McAllen, TX USA; 2 Graduate Program in Biochemistry and Molecular Biology, University of Texas Rio Grande Valley, Edinburg, TX USA; 3 School of Medicine, University of Texas Rio Grande Valley, Edinburg, TX USA; 4 Department of Health & Biomedical Sciences, College of Health Professions, University of Texas Rio Grande Valley, Edinburg, TX USA; 5 Division of Biological Science and Technology, Yonsei University, Wonju, 26493 Republic of Korea; 6 South Texas Center for Excellence in Cancer Research, University of Texas Rio Grande Valley, McAllen, TX USA; 7 College of Pharmacy, The Catholic University of Korea, Bucheon-si, Gyeonggi-do, 14662 Republic of Korea; 8 BK21-4TH, and RCD Control Material Research Institute, College of Pharmacy, The Catholic University of Korea, Bucheon-si, Gyeonggi-do, 14662 Republic of Korea; 9 Present Address: Graduate Program in Cancer Biology, University of Texas Southwestern Medical Center, Dallas, TX USA; 10 Present Address: Biopharmaceutical Research Center, Ochang Institute of Biological and Environmental Science, Korea Basic Science Institute, Cheongju-si, 28119 Republic of Korea

**Keywords:** Squamous cell carcinoma, Macroautophagy

## Abstract

Ultraviolet B (UVB) radiation can distort cellular homeostasis and predispose the skin to carcinogenesis. Amongst the deteriorating effects of the sun’s UVB radiation on cellular homeostasis is the formation of DNA photoproducts. These photoproducts can cause significant changes in the structure and conformation of DNA, inducing gene mutations which may accumulate to trigger the formation of skin cancer. Photoproducts are typically repaired by nucleotide excision repair. Notwithstanding, when the repair mechanism fails, apoptosis ensues to prevent the accumulation of mutations and to restore cellular homeostasis. This present study reports that T-cell protein tyrosine phosphatase (TC-PTP) can increase UVB-induced apoptosis by inhibiting autophagy-mediated cell survival of damaged keratinocytes. TC-PTP deficiency in 3PC mouse keratinocytes led to the formation of autophagic vacuoles and increased expression of LC3-II. We established human TC-PTP-deficient (TC-PTP/KO) HaCaT cells using the CRISPR/Cas9 system. TC-PTP/KO HaCaT cells exhibited increased cell survival upon UVB exposure, which was accompanied by increased expression of LC3-II and decreased expression of p62 compared to control cells. Pretreatment of TC-PTP/KO HaCaT cells with early-phase autophagy inhibitor, 3-methyladenine significantly decreased the expression of LC3-II and reduced cell survival in response to UVB irradiation in comparison with untreated TC-PTP/KO cells. Pretreatment of TC-PTP/KO HaCaT cells with late-phase inhibitor, chloroquine also significantly reduced cell viability with increased accumulation of LC3-II after UVB irradiation compared to untreated counterpart cells. While UVB significantly increased apoptosis in the engineered (Mock) cells, this was not observed in similarly treated TC-PTP/KO HaCaT cells. However, chloroquine treatment increased apoptosis in TC-PTP/KO HaCaT cells. Examination of human squamous cell carcinomas (SCCs) revealed that TC-PTP expression was inversely correlated with LC3 expression. Our findings suggest that TC-PTP negatively regulates autophagy-mediated survival of damaged cells following UVB exposure, which can contribute to remove damaged keratinocytes via apoptosis.

## Introduction

Autophagy is a conserved cellular activity that contributes to the maintenance of homeostasis through the degradation and recycling of intracellular components to ensure the production of energy and the recycling of nutrients for cell survival. Autophagy helps avoid the buildup of damaged organelles and proteins that could be hazardous to the cell, so it is regarded as the quality control system of cells [1, 2]. This mechanism utilizes double-membrane vesicles known as autophagosomes to deliver the components to be degraded into lysosomes, where the recycling occurs. Autophagy becomes more active in response to increased energy demands, stressful conditions, or the accumulation of damaged components in the cell [3, 4].

Protein tyrosine phosphatases (PTPs) play a key role in modulating phosphotyrosine signaling. Specifically, its rate and duration within cells and thus, their regulation has been implicated in carcinogenesis, just like the extensively studied protein tyrosine kinases [5, 6]. T-cell protein tyrosine phosphatase (TC-PTP) is an intracellular and nonreceptor PTP that gets stimulated when the skin is exposed to ultraviolet B (UVB) irradiation [7, 8]. It is encoded by the PTPN2 gene, which gets spliced during its expression into two isoforms, namely TC45 and TC48. The expression of TC-PTP has been widely reported in adult and embryonic tissues where they regulate important functions and has thence incited the design of targeted therapeutics [9–11]. Studies performed using TC-PTP knockout mice showed its critical role in hematopoiesis and immune function because TC-PTP knockout mice were severely defective in the hematopoietic compartment and all homozygous mice died between 3 and 5 weeks of age due to diarrhea, splenomegaly, lymphadenopathy, and anemia [12]. TC-PTP is also involved in the regulation of diabetes and obesity through its ability to modulate insulin and leptin signaling [13–15]. In terms of cancer, TC-PTP may be considered an oncogene or a tumor suppressor depending on cellular context and cancer types being discussed. Studies revealed that TC-PTP can suppress tumor growth in several types of cancers, including breast cancer and hepatocellular carcinoma [16–19]. In contrast, recent studies showed TC-PTP contributes to immunotherapy resistance by reducing IFNγ signaling [20]. Thus, the exact role of TC-PTP and its mechanism in cancer needs to be unraveled for its therapeutic application.

Stimulatory responses of the skin to UVB irradiation include the upregulation of signaling pathways that support survival, clonal expansion, and sustained proliferation of UVB-damaged cells into malignant cells. Some of these pathways include c-Jun N-terminal kinase (JNK), signal transducer and activator of transcription 3 (STAT3), and AKT signaling amongst several others [8, 21]. TC-PTP downregulates these UVB-mediated activated pathways, which results in the removal of damaged keratinocytes by upregulating the apoptotic program and suppressing the proliferation of damaged cells. STAT3 signaling has been implicated in the survival and proliferation of UVB-damaged keratinocytes leading to skin carcinogenesis [22, 23]. Previous studies have shown that TC-PTP-deficient immortalized primary keratinocytes express significantly higher levels of phosphorylated STAT3 which is paralleled by increased proliferation of cells when compared to wildtype counterparts. It has been demonstrated that TC-PTP gets activated following irradiation with UVB and mediates STAT3 dephosphorylation, thus deactivating the STAT3 signaling pathway and preventing UVB-induced skin cancer formation. Likewise, downregulation of STAT3, JNK, and AKT signaling by TC-PTP increases the susceptibility of cells to apoptosis and this evidence reiterates the indispensable role of TC-PTP in the prevention of skin carcinogenesis [7, 24–28].

In skin carcinogenesis induced by UVB irradiation, DNA-damaged cells undergo apoptosis mainly through the mitochondrial apoptotic pathway upon exposure to UVB. Signaling pathways involved in skin tumor initiation modulate the mitochondrial pathway by regulating the expression of either pro- or anti-apoptotic proteins of the Bcl-2 family. Most studies of tumor initiation have focused on identifying the role of these signaling pathways in the regulation of Bcl-2 family proteins [29–31]. From the studies of tumor initiation, it has become clear that autophagy is one critical process involved in tumor initiation through its crosstalking with apoptosis [32, 33]. Autophagy can induce cell death by either cooperating with the apoptotic pathway or promoting it. This current study seeks to report on the regulatory role of TC-PTP on UVB-induced autophagy as a cell survival mechanism in human keratinocytes.

## Results

### TC-PTP deficiency in mouse keratinocytes increases autophagy

**Fig. 1:**
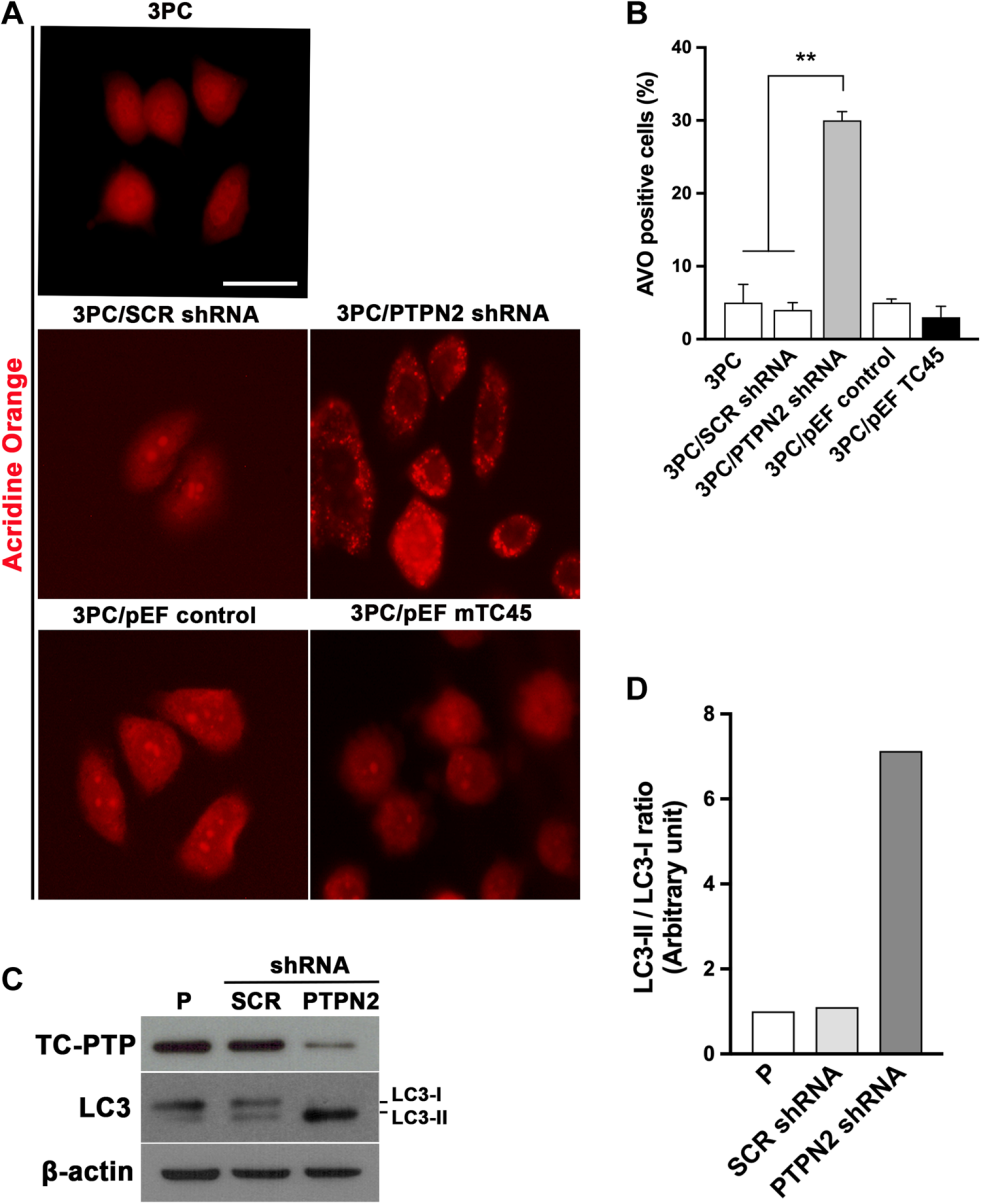
TC-PTP deficiency increases autophagy in mouse keratinocytes. **A**, **B** Parental, control, TC-PTP-deficient, and overexpressing 3PC cells were cultured and then stained with acridine orange. **A** Representative images of cells stained with acridine orange. Scale bar: 20 μm. **B** Quantitative analysis of the number of AVOs. **C** Immunoblot analysis of LC3 expression. Parental, control (scrambled shRNA), TC-PTP-deficient 3PC cells were cultured, and total cell lysates were prepared. **D** Quantitative analysis of LC3-II to LC3-I relative to the β-actin control.

Our previous studies showed that TC-PTP can facilitate UVB-induced apoptosis by negatively regulating STAT3 and fetal liver kinase-1 (Flk-1)/JNK-dependent cell survival signaling [7, 24]. With this regard, loss of epidermal TC-PTP in mice led to increased resistance in UVB-induced apoptosis. Damaged cell death during tumor initiation is tightly modulated by the crosstalk between apoptosis and autophagy. To investigate the impact of TC-PTP in autophagy, TC-PTP-deficient (3PC/PTPN2 shRNA) and TC-PTP-overexpressing (3PC/pEF mTC45) mouse keratinocytes that we previously established [7] were cultured and stained with acridine orange to evaluate the autophagic flux. The formation of acidic vesicular organelles, one of the characteristic features of autophagy, was significantly increased in TC-PTP-deficient cells compared to wildtype (3PC) or control (3PC/SCR shRNA) cells. Their formation was not changed by TC-PTP overexpression, and the number of acidic vesicular organelles was similarly observed between control (3PC/pEF control) and TC-PTP-overexpressing cells (Fig. 1A, B). To further investigate the involvement of TC-PTP in the regulation of autophagy, we examined the conversion of autophagic marker microtubule-associated protein 1 light chain 3-I (MAP1LC3/LC3-1) to LC3-II which occurs during autophagosome formation [34]. Western blot analysis showed an increase in the ratio of LC3-II to LC3-I in TC-PTP-deficient cells compared to wildtype and control cells (Fig. 1C, D), confirming that TC-PTP deficiency can increase the autophagic flux in mouse keratinocytes even in the absence of stimuli, such as UVB irradiation.

### Establishment and characterization of HaCaT TC-PTP knockout keratinocytes

**Fig. 2:**
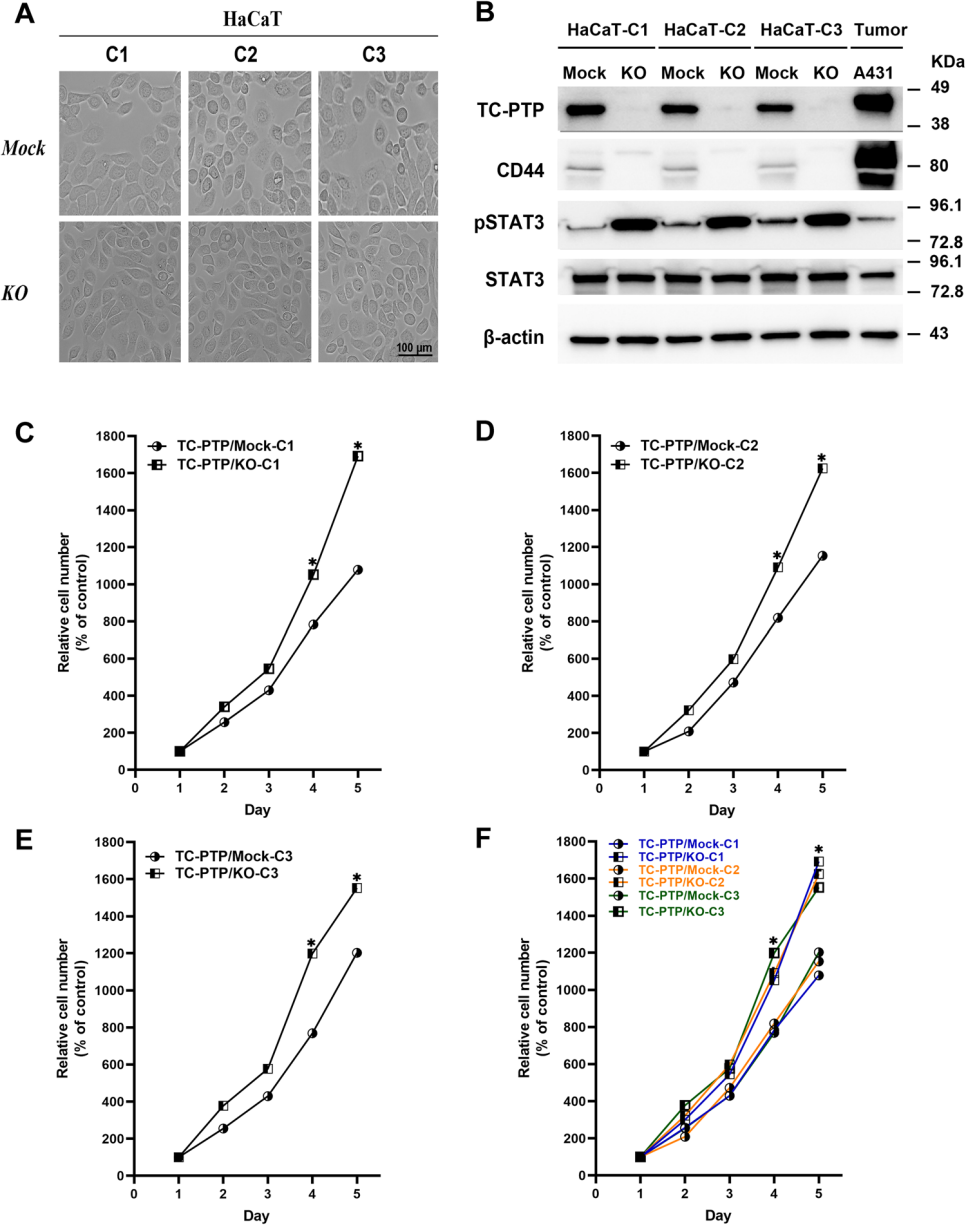
Generation and characterization of HaCaT TC-PTP knockout (KO) keratinocytes. TC-PTP/Mock (engineered control) and HaCaT TC-PTP/KO cells were generated using the CRISPR/Cas9 genome editing system. **A** Representative photomicrographs of HaCaT TC-PTP/KO and TC-PTP/Mock cells after 3 days of culture. All three clones selected for both cell lines were cultured under the same conditions. **B** Immunoblot analysis of HaCaT TC-PTP/Mock and TC-PTP/KO cell lysates with antibodies specific for TC-PTP, CD44, pSTAT3, STAT3, and β-actin. A431 epidermoid carcinoma cell line was used as a positive control. **C**–**F** Cell proliferation of the selected clones (C1–C3) of HaCaT TC-PTP/Mock and TC-PTP/KO cells. Proliferation of the cells was measured using WST-8 assay according to the manufacturer’s general manual. The results are the mean ± standard deviation from three independent experiments. *P < 0.005 by *T*-test for equality of means. **C** HaCaT clones C1. **D** HaCaT clones C2. **E** HaCaT clones C3. **F** HaCaT clones C1–C3.

Mouse skin is different from human skin, even though transgenic mouse models are extensively used to study skin diseases including skin cancer. For example, there are structural differences between mouse and human skin. Structural comparisons between mouse and human skin reveal that the epidermis and dermis are thicker in the human skin in comparison to the mouse skin [35]. Our previous study also showed that TC-PTP is expressed to a greater extent in the nucleus of human keratinocytes compared to mouse keratinocytes [36]. To further investigate the role of TC-PTP in the regulation of autophagy in human keratinocytes, we established HaCaT TC-PTP knockout (KO) cells using the CRISPR/Cas9 genome editing system as described in Materials and Methods. Three clones (HaCaT -C1, C2, and C3) were selected from each genotype. There were no observable variations with respect to morphological characteristics amongst the three clones (HaCaT C1–C3) for each cell line (Fig. 2A). With reference to previous studies demonstrating the positive correlation between the knockdown of TC-PTP and phosphorylated STAT3 expression in both mice and immortalized primary keratinocytes [24, 26], we probed the expression levels of STAT3 in all the clones of HaCaT cell lines. Consistent with previous reports, phosphorylated STAT3 expression was significantly higher in all clones of the TC-PTP/KO cells as compared to all clones of the engineered control (Mock) cells (Fig. 2B). Constitutive activation of STAT3 is known to induce cellular transformation in immortalized fibroblasts [37]. Keratinocytes positive for CD44 expression are known to exhibit enhanced colony formation. It is also reported that direct binding of STAT3 with CD44 and NF-kB increases CD44 expression via human telomerase reverse transcriptase-mediated autocrine signaling in the breast cancer cell lines, which can contribute to promote a cancer stem cell phenotype [38]. To evaluate the anchorage-independent potential of HaCaT cell clones, the expression of the epidermal stem cell marker, CD44, was probed with a specific antibody. A431 epidermoid carcinoma cell line was used as a positive control. As shown in Fig. 2B, there was no CD44 expression for all HaCaT clones indicating their inability to form colonies. Cell viability assay using all clones of both HaCaT Mock and KO cells also showed that akin to reports in previous experiments, HaCaT Cas9/TC-PTP KO clones grew significantly faster than their mock counterparts. Relative cell numbers as a percentage of the control for all clones were comparable for each cell line (Fig. 2C–F). Owing to the observable similarities in morphology and growth amongst the clones, we chose clone 1 (C1) of each of the cell lines and cultured them under the same conditions for all other experiments.

### TC-PTP deficiency in HaCaT cells leads to decreased apoptosis and increased autophagy following UVB irradiation

**Fig. 3:**
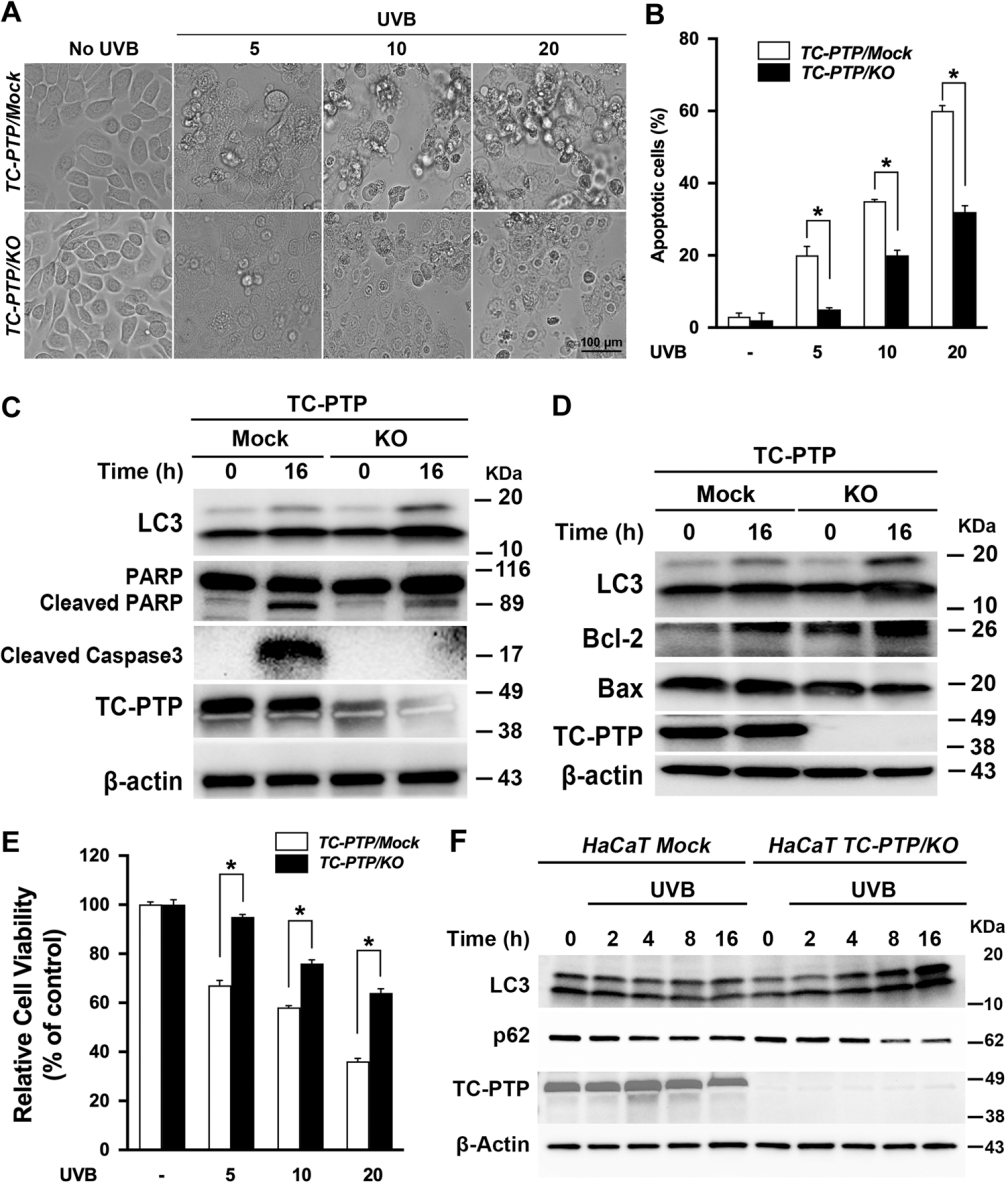
TC-PTP deficiency decreases apoptosis and increases autophagy in human HaCaT keratinocytes following UVB exposure. **A**, **B** HaCaT TC-PTP/Mock and TC-PTP/KO cells were exposed to 5, 10, or 20 mJ/cm^2^ of UVB irradiation and incubated for 16 h following UVB exposure. **A** Representative photomicrographs of HaCaT TC-PTP/Mock and TC-PTP/KO cells after UVB exposure. Scale bar: 100 μm. **B** Quantitative analysis of the percentage of apoptotic cells characterized by cell ballooning, nuclear condensation, and bleb formation. After 16 h of UVB treatment, apoptotic keratinocytes were counted microscopically in at least three non-overlapping fields. Results are the mean ± standard deviation from three independent experiments. *P < 0.05 by *T*-test for equality of means. **C**, **D** HaCaT TC**-**PTP/Mock and TC-PTP/KO cells exposed to 10 mJ/cm^2^ of UVB irradiation and cells were harvested after 16 h following UVB exposure. **C** Immunoblot analysis of HaCaT TC-PTP/Mock and TC-PTP/KO cell lysates with antibodies specific for LC3, cleaved PARP, cleaved caspase-3, TC-PTP, and β-actin. **D** Immunoblot analysis of HaCaT TC-PTP/Mock and TC-PTP/KO cell lysates with antibodies specific for LC3, Bcl-2, Bax, TC-PTP, and β-actin. **E** Cell viability was measured using WST-8 assay. *P < 0.005 by *T*-test for equality of means. **F** Immunoblot analysis of HaCaT TC-PTP/Mock and TC-PTP/KO cell lysates with antibodies specific for LC3, SQSTM1, TC-PTP, and β-actin. TC-PTP/Mock and TC-PTP/KO cells were exposed to 10 mJ/cm^2^ of UVB and harvested at the indicated time post-UV irradiation. Total cell lysates were then prepared.

To investigate the impact of TC-PTP deficiency on cell survival in response to UVB exposure, both HaCaT TC-PTP/Mock and TC-PTP/KO cells were cultured and irradiated with UVB. Without UVB exposure, no visible differences in morphology were observed between TC-PTP/Mock and TC-PTP/KO cells. However, following UVB exposure, profound morphological changes caused by the apoptotic response, including membrane blebbing and cellular ballooning, were observed in TC-PTP/Mock keratinocytes. These changes were significantly reduced in the absence of TC-PTP (Fig. 3A, B), which is consistent with previous studies performed using mouse TC-PTP-deficient keratinocytes. Western blot analysis showed that the levels of two apoptotic markers, cleaved poly[ADP-ribose] polymerase (PARP) and cleaved caspase-3, were significantly lower in HaCaT TC-PTP/KO cells compared to HaCaT TC-PTP/Mock cells (Fig. 3C). In addition, the level of anti-apoptotic Bcl-2 expression was higher in HaCaT TC-PTP/KO cells compared to HaCaT TC-PTP/Mock cells after UVB irradiation. In contrast, the expression of pro-apoptotic Bax was lower in HaCaT TC-PTP/KO cells in comparison with HaCaT TC-PTP/Mock cells (Fig. 3D). Similar to this observation, cell viability in TC-PTP/Mock cells was significantly decreased after UVB irradiation in a dose-dependent manner as compared to TC-PTP/KO cells (Fig. 3E). To investigate whether the enhanced cell survival observed in TC-PTP/KO cells after UVB is associated with increased autophagy, we examined the expression of two specific markers of autophagy, LC3 and SQSTM1/p62 (sequestosome 1), using western blot analysis. The ratio of the conversion of LC3-I to LC3-II was significantly increased with increased level of LC3-II protein production in HaCaT TC-PTP/KO cells following UVB exposure in a time-dependent manner compared with HaCaT TC-PTP/Mock cells (Fig. 3F). The ubiquitin-binding scaffold protein SQSTM1 is known to bind directly and form a complex with LC3 and then lead to targeted degradation of SQSTM1-associated polyubiquitin-containing proteins by autophagy. When autophagy is induced, the level of SQSTM1 expression is decreased, while inhibition of autophagy can cause SQSTM1 to accumulate [39, 40]. In addition to increased production of LC3-II expression in TC-PTP/KO cells following UVB, the level of SQSTM1 expression was gradually reduced in TC-PTP/KO cells following UVB exposure in a time-dependent manner compared with TC-PTP/Mock cells (Fig. 3F), indicating that TC-PTP deficiency in human keratinocytes increases autophagic response in response to UVB irradiation, which may increase cell survival.

### Inhibition of autophagy decreases cell survival of HaCaT TC-PTP/KO cells after UVB exposure

**Fig. 4:**
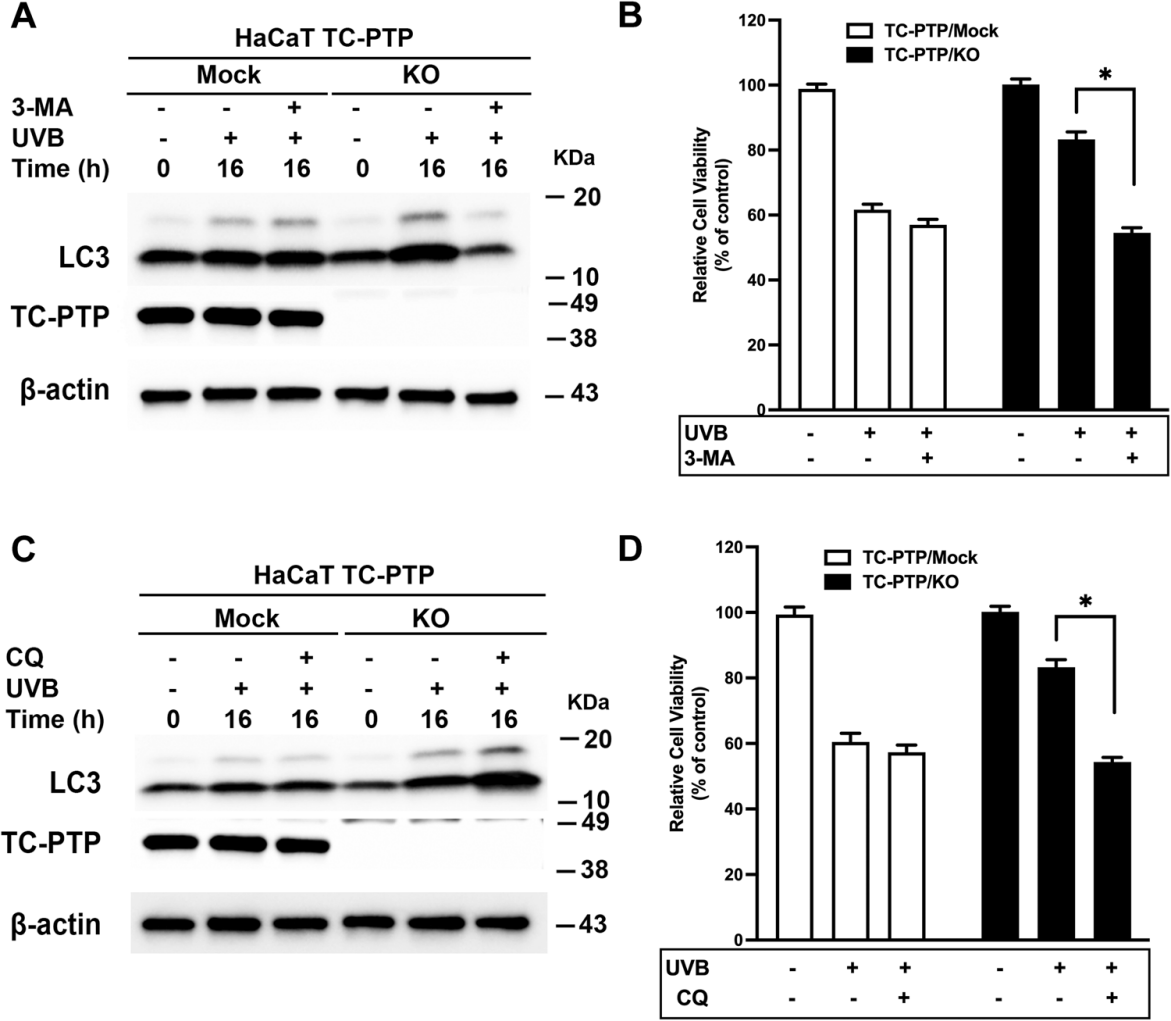
Inhibition of autophagy in keratinocyte survival and proliferation following UVB exposure. **A**, **B** Effect of inhibition of autophagy on keratinocyte survival and proliferation in response to UVB irradiation. TC-PTP/Mock and TC-PTP/KO cells were pretreated with 3-MA (5 mM) for 1-h prior exposure to UV irradiation (10 mJ/cm^2^). Cells were then collected 16 h after UVB irradiation. **A** Immunoblot analysis of HaCaT TC-PTP/Mock and TC-PTP/KO cell lysates with antibodies specific for LC3, TC-PTP, and β-actin. **B** Cell viability of the cells was measured using WST-8 assay. *P < 0.005 by *T-*test for equality of means. **C**, **D** Effect of inhibition of autophagy on keratinocyte survival and proliferation in response to UVB irradiation. TC-PTP/Mock and TC-PTP/KO cells were pretreated with CQ (50 μM) for 1-h prior exposure to UV irradiation (10 mJ/cm^2^). Cells were then collected 16 h after UVB irradiation. **C** Immunoblot analysis of HaCaT TC-PTP/Mock and TC-PTP/KO cell lysates with antibodies specific for LC3, TC-PTP, and β-actin. **D** Cell viability of the cells was measured using WST-8 assay. *P < 0.005 by *T-*test for equality of means.

To evaluate whether UVB-induced autophagy observed in TC-PTP/KO cells contributes to an increase in cell survival, both TC-PTP/Mock and TC-PTP/KO cells were treated with specific autophagy inhibitors before UVB exposure. Western blot analysis showed that the level of LC3-II expression was increased in HaCaT TC-PTP/KO cells in comparison with HaCaT TC-PTP/Mock cells after UVB irradiation. However, pretreatment with 3-methyladenine (3-MA), an early-phase autophagy inhibitor, before UVB exposure significantly reduced the level of LC3-II production in TC-PTP/KO cells (Fig. 4A). With this regard, TC-PTP/KO cells showed a significant decrease in cell viability when they were pretreated with 3-MA prior to UVB irradiation as compared to TC-PTP/KO cells treated with UVB alone. However, relative cell viability for HaCaT TC-PTP/Mock cells was comparable between those pretreated with 3-MA before UVB and those treated with UVB alone (Fig. 4B). Inhibition of late-phase autophagy by chloroquine (CQ) increases the level of LC3-II production by impairing autophagosome fusion with lysosome. As expected, the level of LC3-II expression was increased in HaCaT TC-PTP/KO cells in comparison to HaCaT TC-PTP/Mock cells after UVB irradiation. Pretreatment with CQ before UVB exposure significantly increased the level of LC3-II production in TC-PTP/KO cells compared with only UVB-exposed TC-PTP/KO cells (Fig. 4C). In addition, cell viability was significantly reduced in TC-PTP/KO cells with pretreatment of CQ before UVB as compared to TC-PTP/KO cells treated with UVB alone (Fig. 4D).

**Fig. 5:**
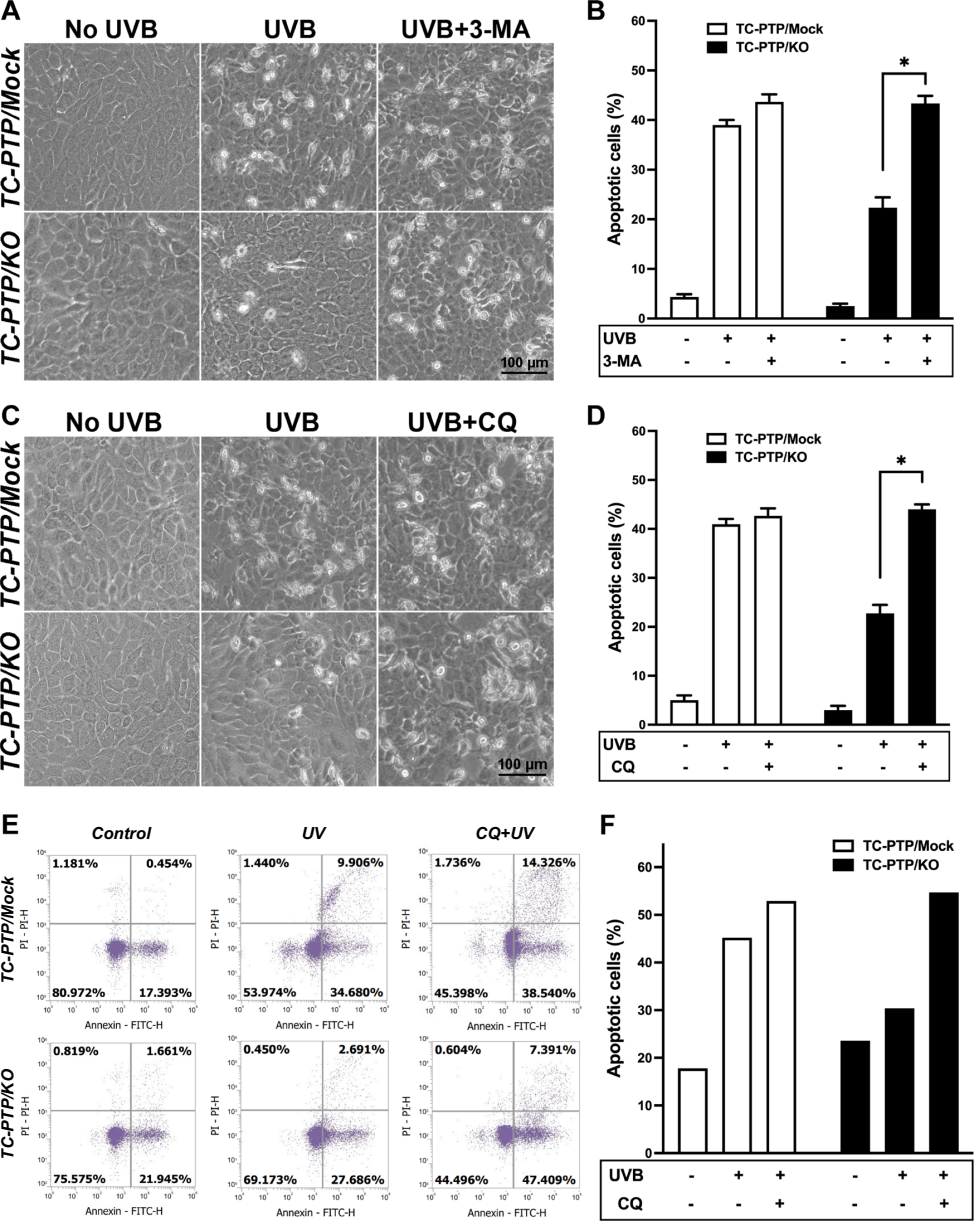
Inhibition of autophagy on the regulation of UVB-induced apoptosis in keratinocytes. **A**–**C** TC-PTP/Mock and TC-PTP/KO cells were pretreated with 3-MA (5 mM) for 1-h prior exposure to UV irradiation (10 mJ/cm^2^). Cells were then collected 16 h after UVB irradiation. **A** Representative photomicrographs of HaCaT TC-PTP/Mock and TC-PTP/KO cells after UVB exposure in the presence or absence of 3-MA. Scale bar: 100 μm. **B** Quantitative analysis of the percentage of apoptotic cells characterized by cell ballooning, nuclear condensation, and bleb formation. After 16 h of UVB treatment, apoptotic keratinocytes were counted microscopically in at least three non-overlapping fields. Results are the mean ± standard deviation from three independent experiments. *P < 0.05 by *T-*test for equality of means. **C**, **D** TC-PTP**/**Mock and TC-PTP/KO cells were pretreated with CQ (50 μM) for 1-h prior exposure to UV irradiation (10 mJ/cm^2^). Cells were then collected 16 h after UVB irradiation. **C** Representative photomicrographs of HaCaT TC-PTP/Mock and TC-PTP/KO cells after UVB exposure in the presence or absence of CQ. Scale bar: 100 μm. **D** Quantitative analysis of the percentage of apoptotic cells characterized by cell ballooning, nuclear condensation, and bleb formation. After 16 h of UVB treatment, apoptotic keratinocytes were counted microscopically in at least three non-overlapping fields. Results are the mean ± standard deviation from three independent experiments. *P < 0.05 by *T*-test for equality of means. **E**, **F** TC-PTP/Mock and TC-PTP/KO cells were pretreated with CQ (50 μM) for 1-h prior to exposure to UV irradiation (10 mJ/cm^2^). Apoptotic cells were stained with Annexin V-FITC and estimated using flow cytometry analysis. **E** Representative outputs of flow cytometry analysis. **F** Quantification of apoptotic cells in control, UVB-treated, and CQ-UVB-treated HaCaT TC-PTP/Mock and KO cells 16 h post-UVB irradiation. The results are the mean ± standard deviation from three independent experiments. *P < 0.05 by *T*-test for equality of means.

To further confirm that TC-PTP promotes UVB-induced apoptosis by suppressing autophagy in keratinocytes, both TC-PTP/Mock and TC-PTP/KO cells were pretreated with either 3-MA or CQ before UVB exposure, and morphological changes were examined. Morphological changes caused by apoptotic response were increased in TC-PTP/KO cells treated with 3-MA before UVB exposure, in comparison with those exposed only to UVB. These changes were comparable to those observed in their TC-PTP/Mock counterparts (Fig. 5A, B). Similar to the results obtained using 3-MA, morphological changes caused by apoptotic response were also increased in TC-PTP/KO cells pretreated with CQ before UVB exposure as compared with only UVB-exposed TC-PTP/KO cells (Fig. 5C, D). Similar to these observations, apoptotic cells detected by flow cytometry analysis were significantly higher in HaCaT TC-PTP/Mock cells compared to that in HaCaT TC-PTP/KO cells following irradiation with UVB (Fig. 5E, F). However, when cells were pretreated with CQ prior to UVB irradiation, apoptotic cells were significantly increased in HaCaT TC-PTP/KO cells in comparison to only UVB-exposed TC-PTP/KO cells. Increased apoptotic response was not observed in TC-PTP/Mock cells by CQ pretreatment before UVB exposure. The percentage of apoptotic cells in TC-PTP/KO cells increased by CQ pretreatment was comparable with that of TC-PTP/Mock cells in the absence or presence of CQ pretreatment (Fig. 5E, F). These results confirm that TC-PTP-mediated inhibition of autophagy is critical in the induction of apoptosis in human keratinocytes in response to UVB irradiation.

### LC3 expression is increased in human skin tumors

**Fig. 6:**
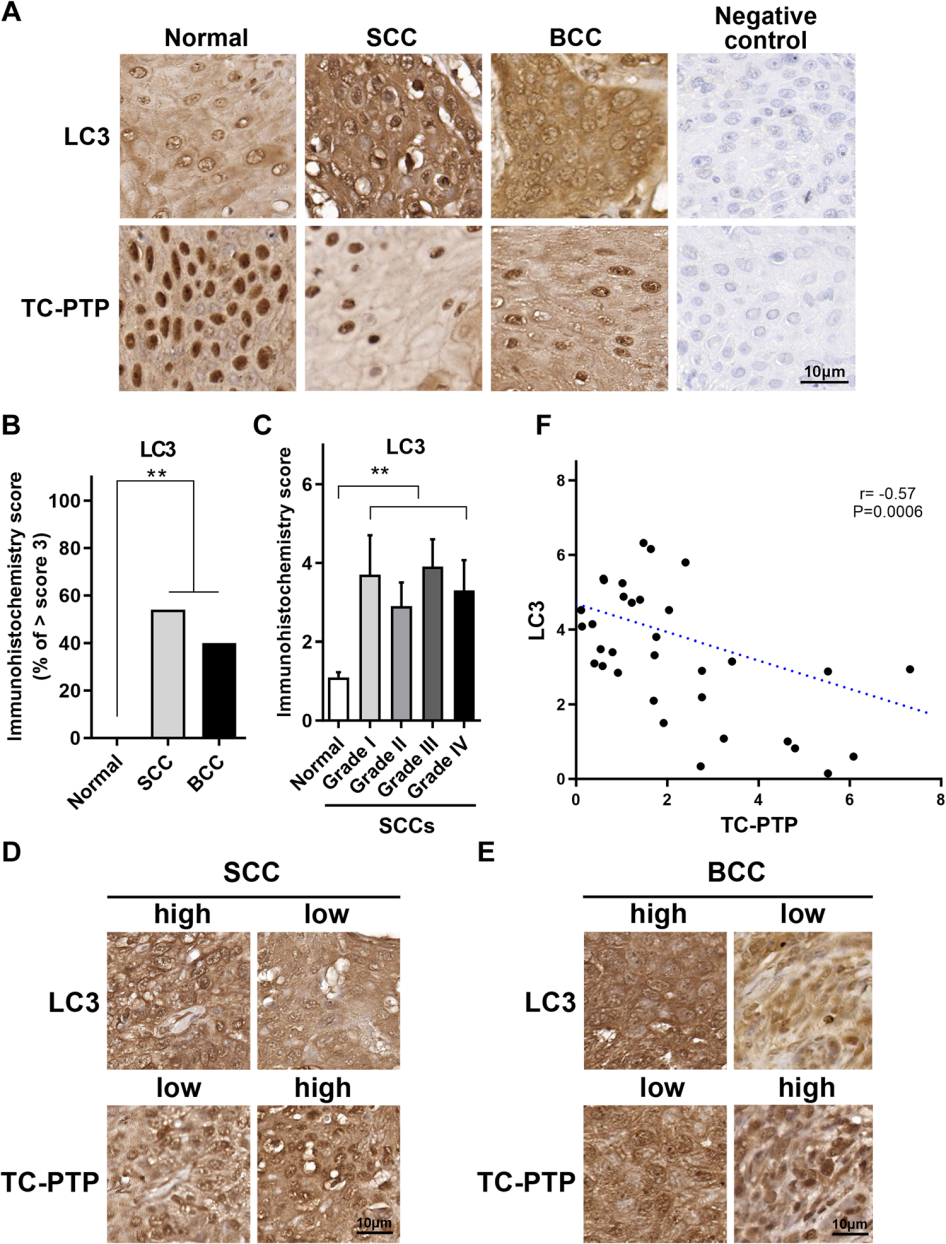
LC3 and TC-PTP expressions in human skin tumors. **A** Representative immunohistochemical staining of LC3 and TC-PTP in normal human skins (*n* = 8), squamous cell carcinomas (SCCs) (*n* = 33), and basal cell carcinomas (BCCs) (*n* = 5). Scale bar: 10 µm. **B** Quantification of LC3 expression by immunohistochemistry (IHC) scoring. Percentage of cells showing more than IHC score 3. LC3 expression levels from each section were assessed as described in Methods. **P < 0.01 by Mann–Whitney *U*-test. **C** Quantification of LC3 expression by immunohistochemistry scoring in different grades of SCCs. **P < 0.01 by Mann–Whitney *U*-test. **D** Representative immunohistochemical staining of LC3 and TC-PTP in SCCs. Scale bar: 10 µm. **E** Representative immunohistochemical staining of LC3 and TC-PTP in BCCs. Scale bar: 10 µm. **F** Correlation scatter plots for expression of LC3 and TC-PTP in human skin tumors. The Pearson correlation coefficient was calculated with a two-tailed *P* value. *r* = −0.57, *P* < 0.0006.

We previously showed that the level of TC-PTP expression was decreased in human squamous cell carcinomas (SCCs) compared to normal human skin sections [25]. To investigate whether TC-PTP expression is associated with the suppression of autophagy in human skin cancers, the levels of TC-PTP and LC3 expression were analyzed in normal skin tissues and skin cancer tissues by immunohistochemical analysis. While TC-PTP expression was significantly decreased in both SCCs (*n* = 35) and basal cell carcinomas (BCCs) (*n* = 5) in comparison with normal human skin sections (*n* = 9), LC3 expression was significantly increased in both SCCs and BCCs compared to normal skin (Fig. 6A, B). LC3 expression was significantly higher in all grades of human SCCs investigated, regardless of the tumor grade (Fig. 6C). Further analysis showed that cells expressing high levels of TC-PTP express low levels of LC3 in both SCCs and BCCs. In contrast, cells expressing low levels of TC-PTP express high levels of LC3 in both SCCs and BCCs (Fig. 6D, E). Consistent with this observation, quantification of staining showed that the expression of LC3 was significantly (*P* < 0.0006) inversely correlated with TC-PTP expression (Fig. 6F). These findings further confirm that enhanced autophagy due to the loss of TC-PTP may play a critical role in skin tumor formation.

## Discussion

Autophagy plays critical roles in maintaining the homeostasis of keratinocytes by regulating various physiological functions including survival, proliferation, differentiation, and senescence. Autophagy can function as an alternative cell death mechanism, particularly within apoptosis-deficient cells, thereby, contributing to tumor suppression [41, 42]. Autophagy activation contributes to intracellular remodeling during keratinocyte differentiation by mediating the degradation of cellular components including cytosolic proteins. It suggests that autophagy-induced differentiation may contribute to inhibiting proliferation [43, 44]. Conversely, autophagy can also inhibit cell death as a cell survival mechanism that is induced by environmental stresses, including nutrient deficiency, chemotherapy, radiation, and hypoxia [41, 42, 45, 46]. With this regard, the loss of GABARAP, a crucial protein in autophagosome formation, suppresses DMBA-induced tumorigenesis by inducing apoptosis [47]. UV radiation was shown to induce autophagy which resulted in the promotion of inflammation and skin carcinogenesis [48]. UV-induced autophagy also was able to protect keratinocytes against apoptosis [49], suggesting that autophagy might function as a cytoprotective mechanism against environmental stress-induced apoptosis in keratinocytes. Our current studies show that autophagy is significantly increased in TC-PTP/KO keratinocytes in response to UVB exposure, implying autophagy can block apoptosis in the absence of TC-PTP during tumor initiation.

Autophagy was significantly higher in TC-PTP-deficient mouse keratinocytes in the absence of UVB irradiation compared to wildtype and TC-PTP-overexpressing keratinocytes, as demonstrated by an increased ratio of the conversion of LC3-I to LC3-II in TC-PTP-deficient keratinocytes (Fig. 1C, D). In contrast, this increased conversion was not observed in human TC-PTP/KO keratinocytes in the absence of UVB exposure as compared to TC-PTP/Mock keratinocytes. However, UVB exposure significantly increased the level of LC3-II production and decreased the level of SQSTM1 expression in TC-PTP/KO keratinocytes in a time-dependent manner. This was not observed in TC-PTP/Mock keratinocytes (Fig. 3F), indicating that TC-PTP prevents UVB-mediated autophagic response. Inhibition of autophagy by either 3-MA or CQ before UVB exposure significantly reduced cell viability in TC-PTP/KO keratinocytes in comparison to untreated controls. However, relative cell viability in TC-PTP/Mock cells was comparable regardless of pretreatment with autophagy inhibitors (Fig. 4B, D). Pretreatment of CQ before UVB exposure significantly increased annexin V-positive apoptotic cells in TC-PTP/KO cells to a level comparable with that in TC-PTP/Mock cells (Fig. 5E, F). These results suggest that TC-PTP-mediated downregulation of autophagy leads to increased UVB-induced apoptosis in human keratinocytes, which can contribute to the attenuation of skin cancer formation. With this regard, LC3 expression was significantly increased in human SCCs and BCCs, with an accompanying decrease in the expression of TC-PTP in both types of skin cancer as compared to normal skin (Fig. 6A–C). LC3 expression patterns were inversely proportional to TC-PTP expression in skin tumor sections (Fig. 6D–F).

Cell survival via autophagy is regulated by several signaling pathways [50]. The RTK/PI3K/AKT signaling pathway can promote autophagy by activating the RAF1/MEK1/ERK1/2 pathway, although it also can inhibit autophagy by activating the TSC/mTOR pathway [51–53]. Our previous studies showed that TC-PTP deficiency in mouse keratinocytes confers resistance to tumor initiator 7,12-dimethylbenz[a]anthracene (DMBA)-induced apoptosis accompanied by increased expression of phosphorylated AKT. Inhibition of AKT significantly increased cellular sensitivity to DMBA-induced apoptosis in TC-PTP-deficient keratinocytes compared to control keratinocytes [26]. Similar to these results, the level of phosphorylated AKT was significantly increased in TC-PTP/KO HaCaT keratinocytes after UVB exposure compared to TC-PTP/Mock HaCaT keratinocytes (data not shown), implying that TC-PTP-mediated regulation of AKT signaling may contribute to the downregulation of autophagy in response to UVB irradiation.

Our recent studies have shown that the Flk-1/JNK signaling pathways promote keratinocyte cell survival following exposure to UVB, and these pathways can be negatively regulated by TC-PTP. TC-PTP KO keratinocytes showed increased resistance to UVB-induced apoptosis compared with control keratinocytes, and this corresponded with an increase in the level of Flk-1 and JNK phosphorylation. Treatment with either Flk-1 inhibitors or a JNK inhibitor reduced cell survival in TC-PTP KO cells [24]. BNIP3 (Bcl-2 and adenovirus E1B 19-kDa interacting protein 3), a member of the Bcl-2 homolog 3-only subfamily of the Bcl-2 family proteins, can also induce autophagy, although whether this response leads to cell death or survival is still controversial [54–56]. Recent studies have shown that BNIP3-induced autophagy occurs *via* UVB-mediated JNK and ERK/MAPK activation and this mechanism can contribute to the protection of keratinocytes from UVB-induced apoptosis [57]. In addition, our studies have shown that TC-PTP deficiency in keratinocytes reduced epidermal apoptosis induced by UVB through the dephosphorylation of STAT3 [7, 26]. STAT3 is also implicated in multiple aspects of the autophagic process, and it either induces or suppresses autophagy depending on the cellular context [58]. In particular, STAT3 phosphorylation upregulates BNIP3 expression and contributes to the survival of brain cancer cells *via* autophagy [59]. STAT3 activates and stabilizes hypoxia-inducible factor 1α (HIF-1α), which can induce autophagy [60, 61]. Activated HIF-1α further induces BNIP3 expression [62, 63]. Studies have shown that the phosphorylation and subsequent nuclear translocation of STAT3 is induced by VEGF/Flk-1 signaling [64]. STAT3 and HIF-1α increase VEGF expression that is induced by UVB exposure [65–67], and JNK signaling can lead to STAT3 activation [68, 69]. These results suggest that reciprocal regulation of Flk-1/JNK/BNIP3 and STAT3/HIF-1α/BNIP signaling may further increase autophagy during tumor initiation. Further work is needed to assess the impact of inhibiting UVB or DMBA-induced autophagy on keratinocyte survival in TC-PTP KO cells by modulating either Flk-1/JNK/BNIP3 or STAT3/HIF-1α/BNIP3 signaling.

While our studies suggest that autophagy can contribute to enhancing cell survival from UVB-induced apoptosis, studies have shown that autophagy plays a role in maintaining a balance of keratinocyte proliferation and terminal differentiation. During the process of terminal differentiation, autophagy is induced to degrade various subcellular organelles including nucleus and mitochondria, in addition to cytoplasmic proteins, which can contribute to converting metabolically active keratinocytes to inert corneocytes [44]. Also, studies using ATG7-deficient mice showed that autophagy-induced cell death is required for terminal differentiation [70].

In conclusion, UVB-induced DNA damage can either be repaired by DNA repair mechanisms or eliminated by activation of apoptosis. However, in some instances, the damage done to DNA can produce mutation(s) that provide a growth advantage, resulting in a subpopulation of cancerous cells in the initiation stage of skin carcinogenesis. While the loss of TC-PTP in cells led to significantly decreased UVB-induced apoptosis compared to WT cells, TC-PTP deficiency significantly increased autophagy following UVB irradiation at timepoints preceding apoptosis, suggesting that TC-PTP can contribute to apoptotic cell death of mutated keratinocytes by suppressing autophagy. This mechanism may be a crucial contributing factor towards the survival of precancerous cells during tumor initiation. In addition to apoptosis, autophagy regulates epidermal differentiation and senescence and contributes to cellular homeostasis and carcinogenesis. Additional studies using 3D skin models and in vivo mouse models will be helpful to further decipher the functional role of TC-PTP in regulating autophagy in epithelial homeostasis and carcinogenesis against environmental toxicants.

## Materials and methods

### Cell culture and generation of TC-PTP knockout (KO) cell lines

3PC, an immortalized mouse keratinocyte cell line [71], was cultured in a keratinocyte growth medium containing 1% fetal bovine serum until 80–85% confluency. HaCaT keratinocytes (immortalized and nontumorigenic human skin keratinocyte cell line) were cultured in Dulbecco’s Modified Eagle’s Medium (DMEM) containing 10% FBS and 1% penicillin/streptomycin at a temperature of 37 °C and carbon dioxide concentration of 5% until 80–85% confluency. To generate HaCaT TC-PTP/KO cell lines, HaCaT cells were transfected with either the CRISPR/Cas9 plasmid targeting TC-PTP (sc-403071, Santa Cruz Biotechnology) or the corresponding control plasmid (sc-418922, Santa Cruz Biotechnology). For UVB exposure, the cells were washed with prewarmed DPBS and then irradiated with a dose of UVB. The DPBS was aspirated immediately post-UVB irradiation and then culture medium was added to cells. Cells were again maintained under the same incubator conditions until harvest.

### Acridine orange staining

Staining of acidic vesicular organelles (AVOs) by acridine orange (Sigma-Aldrich) was performed according to published procedures [72]. 3PC keratinocytes were plated into a 12-well plate and cultured to reach the desired confluency. Following this, acridine orange (0.5 μg/ml) was added to medium for 30 min. After cells were washed twice with PBS, fluorescence images were acquired with a confocal laser scanning microscope (STELLARIS 8 STED, Leica Microsystems, Germany).

### Cell viability assay

Cell viability was analyzed using a 2-(2-methoxy-4-nitrophenyl)-3-(4-nitrophenyl)-5-(2,4-disulfophenyl)-2*H*-tetrazolium, monosodium salt (WST-8) (Dojindo Laboratories, Japan), which is a highly water-soluble tetrazolium salt that yields a water-soluble formazan dye when reduced by an electron mediator. Briefly, HaCaT cells were plated at a density of 2 × 10^4^ cells/well in a 48-well plate and incubated for 24 h. The cells were washed, coated with DPBS, and then irradiated with 10 mJ/cm^2^ of UVB. After post-UVB incubation for defined hours, the WST-8 dye was added to each well and again incubated per the manufacturer’s instructions. Absorbance was measured at 450 nm.

### Preparation of whole cell lysate and western blot analysis

Total protein lysates from keratinocyte cell lines were prepared using RIPA buffer (Thermo Fisher Scientific) containing 1% Triton X-100, phosphatase inhibitor cocktail I and II, and protease inhibitor cocktail from Sigma-Aldrich. The extracted proteins were quantified using the bicinchoninic acid (BCA) assay and equivalent amounts of the total proteins were resolved using SDS-PAGE. The separated proteins were transferred to a PVDF membrane (GE healthcare) and blocked in 5% BSA or skim milk for either overnight at 4 °C or 1 h at room temperature. The membrane was incubated with a primary antibody overnight at 4 °C, washed in TBST, and then incubated with a horseradish peroxidase-conjugated secondary antibody at room temperature. To detect immunoreactive proteins, the membrane was briefly immersed into a chemiluminescent reagent (Pierce), after which blot images were taken using ChemiDoc^TM^MP (Bio-Rad, Hercules, CA). The following antibodies were utilized: anti-LC3A/B (#4108); anti-LC3B (#3868); anti-phospho-STAT3 (#9145); anti-STAT3 (#9132); anti-SQSTM1/p62 (#5114); and anti-TC-PTP (#58935); anti-Bcl-2 (#4223); anti-Bax (#2772); anti-cleaved caspase-3 (#9664); anti-PARP (#9542) from Cell Signaling Technology; anti-CD44 (#NBP1-47386) from Novus Biologicals and anti-β-Actin (sc-47778) from Santa Cruz Biotechnology.

### Analysis of UVB-induced apoptosis in keratinocytes

Annexin V-FITC apoptosis assay was utilized to analyze UVB-induced apoptosis in keratinocytes. HaCaT cells were plated at a density of 1 × 10^5^ cells/mL in a 100 mm plate and incubated for 24 h. The cells were washed, coated with DPBS, and then irradiated with 10 mJ/cm^2^ of UVB. After post-UVB incubation for defined hours, media with floating cells were collected into a 50 mL falcon tube. The attached cells were trypsinized, collected into the same tube, and centrifuged for 5 min at 1000 RPM. The supernatant was aspirated, and the pellet was resuspended with PBS. The recommended number of cells were collected by centrifugation after counting and resuspended in 1X binding buffer. Both annexin V-FITC and propidium iodide were added. The cells were incubated at room temperature for 5 min in the dark. Annexin V-FITC binding was analyzed by flow cytometry.

### Immunohistochemical analysis

Human skin cancer and normal tissues were purchased from SUPER BIO CHIPS (#CX2). Formalin-fixed and paraffin-embedded tissues were deparaffinized and hydrated using standard procedures. Endogenous peroxidase activity was blocked with 0.03% hydrogen peroxide for 10 min. For the antigen retrieval, sections were microwaved for 5 min in 10 mM sodium citrate buffer (pH 6.0) containing 0.01% Tween 20 and allowed to cool for 20 min. Sections were then stained with anti-TC-PTP (Proteintech, #11214-1-AP), anti-LC3A/B (Proteintech, #14600-1-AP) antibodies following suggested procedures by the manufacturer. All specimens were assessed by H&E staining for morphology. Immunoreactivity was determined by scoring according to the staining intensity (0, none; 1, weak; 2, moderate; 3, strong) of immunolabeling and percent positive cells (0, <5%; 1, 6 to 25%; 2, 26 to 50%; 3, 51 to 75%; 4, 76% to 100%). The final immunoreactive score was calculated by multiplying the positive cell proportion score by the staining intensity score. In the above analysis, there was no discrepancy between the 2 observers regarding the patterns of biomarker expression and the scores assigned to analyzed sections. Data were quantified and analyzed with Image J software.

## Supplementary information

Original data

## Data Availability

The data supporting the results of this study are available by contacting the corresponding author upon request.
