# Supplementary material for: Loss of TC-PTP in keratinocytes leads to increased UVB-induced autophagy
Source: Cell Death Discov. 2025 Feb 28;11:80. doi: 10.1038/s41420-025-02353-8 (PMC11871011; doi:10.1038/s41420-025-02353-8)

Original western blots

Figure 1C

TC-PTP

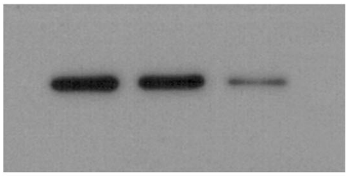

LC3

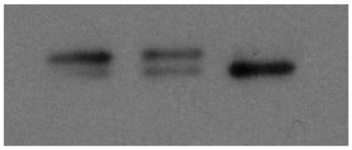

Beta-actin

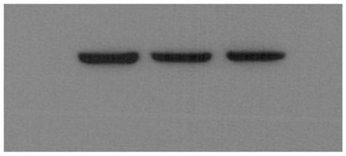

Figure 2B

TC-PTP

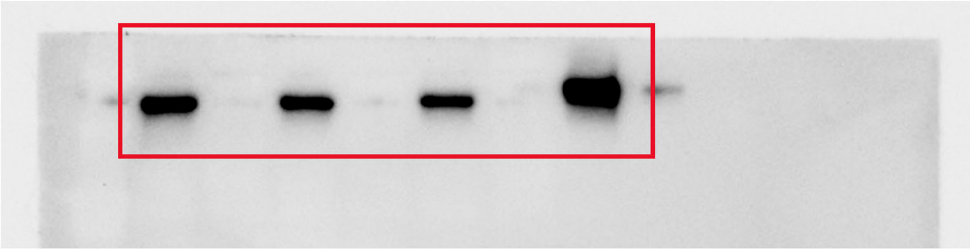

CD44

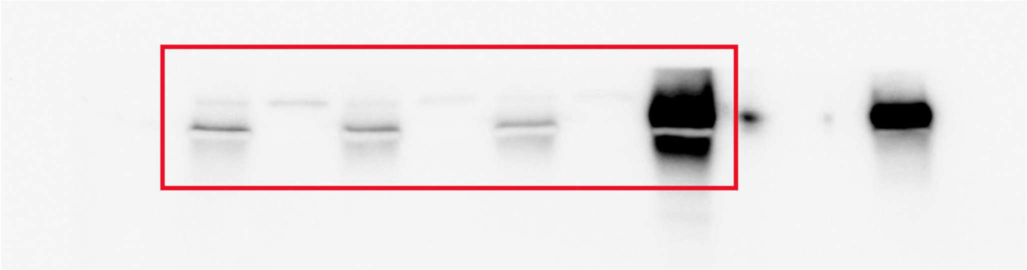

pSTAT3

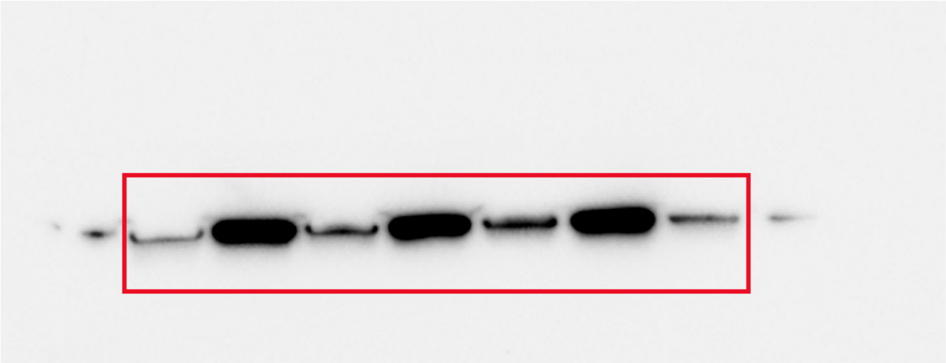

Original western blots

Figure 2B

STAT3

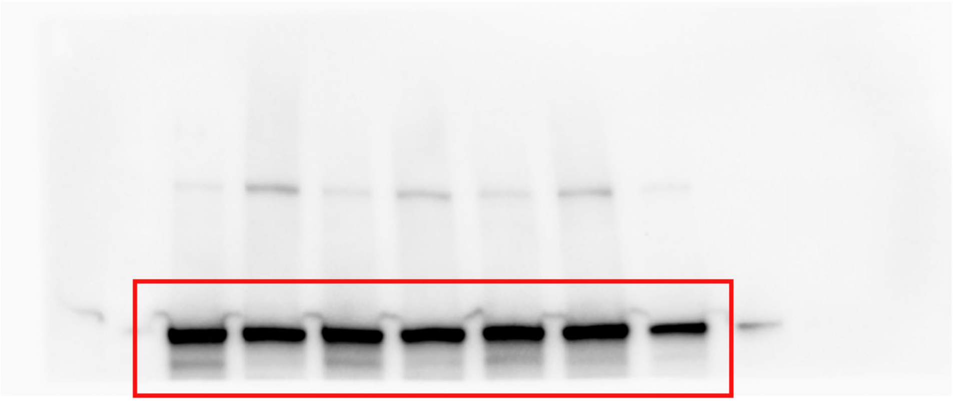

Beta-actin

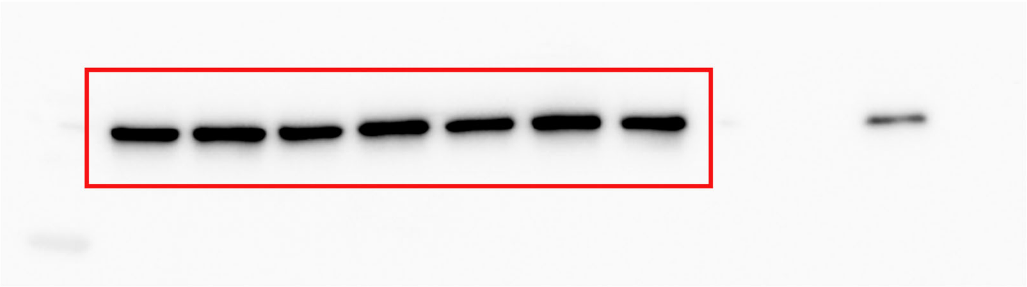

Figure 3C

LC3

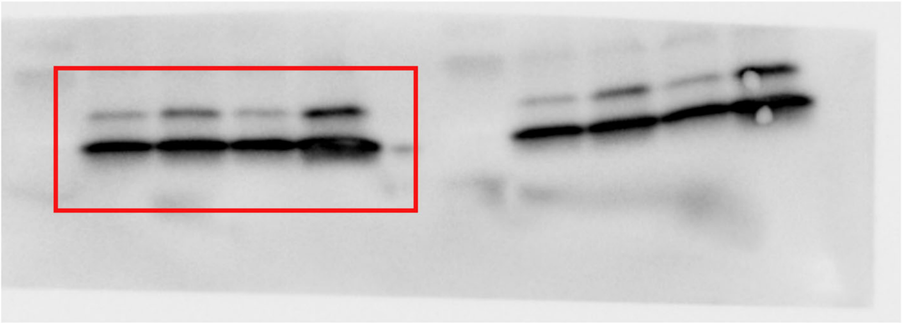

PARP
Cleaved PARP

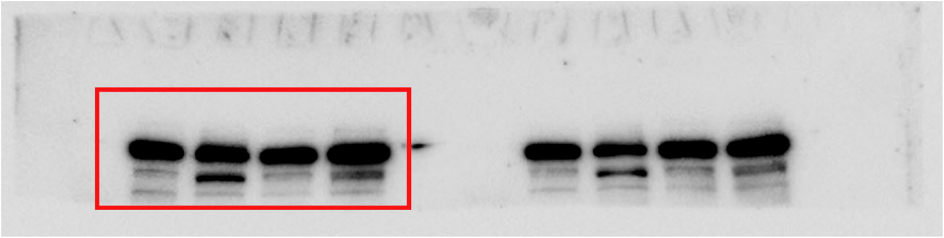

Cleaved Caspase3

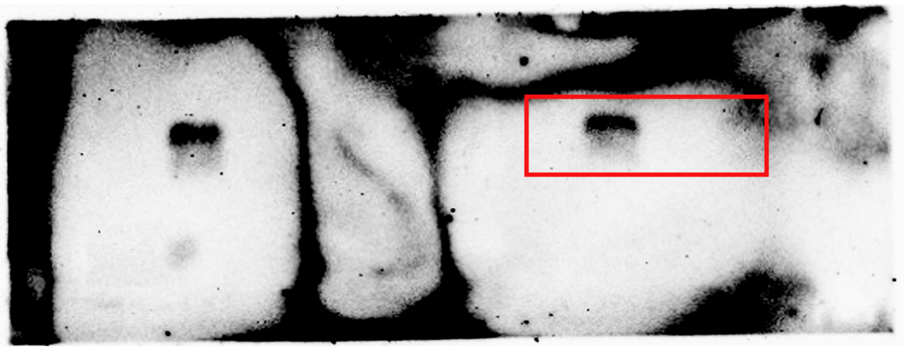

Original western blots

Figure 3C

TC-PTP

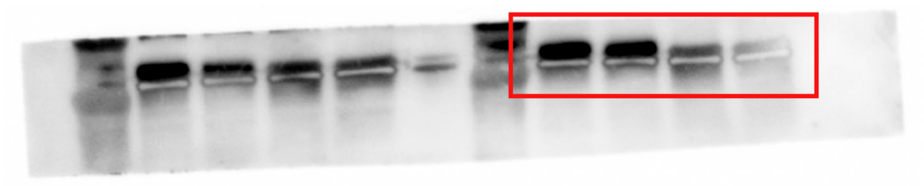

Beta-actin

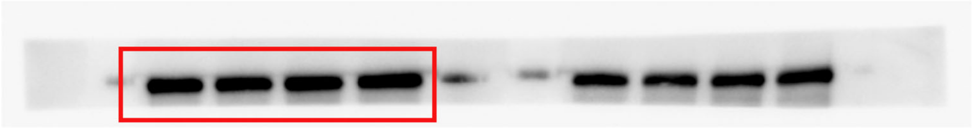

Figure 3D

LC3

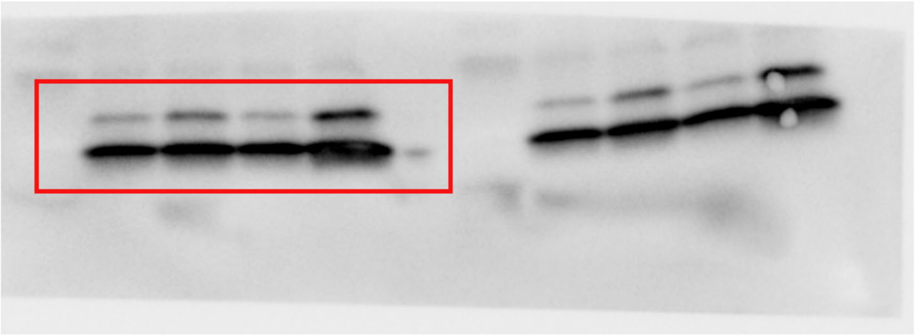

Bcl-2

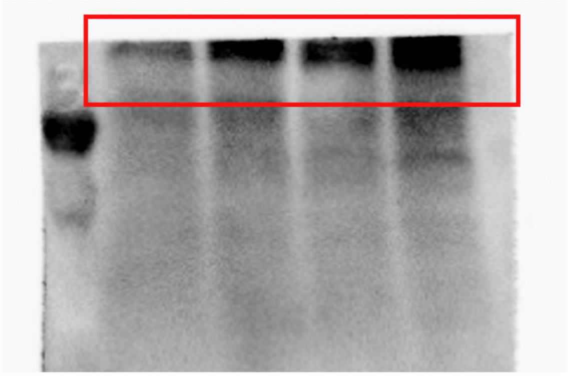

Bax

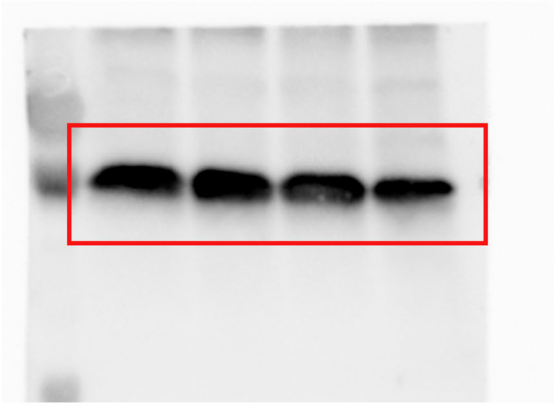

Original western blots

Figure 3D

TC-PTP

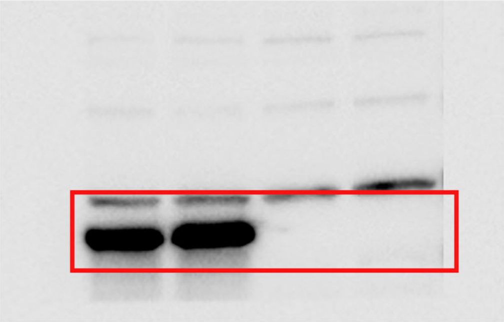

Beta-actin

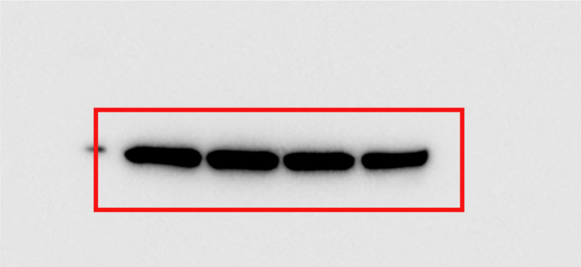

Figure 3F

LC3

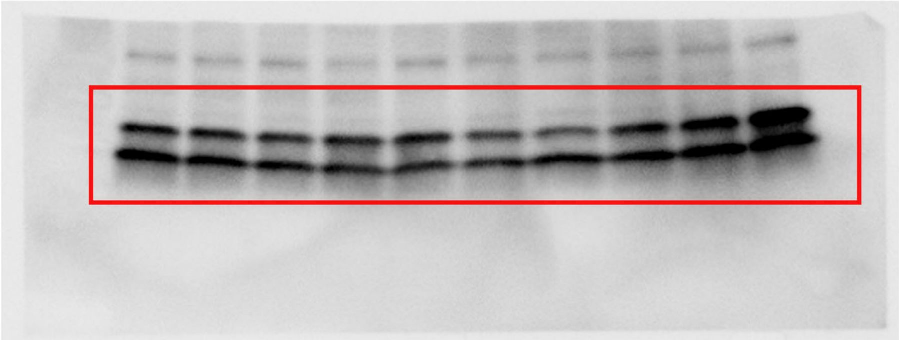

p62

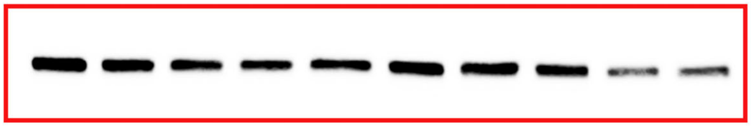

TC-PTP

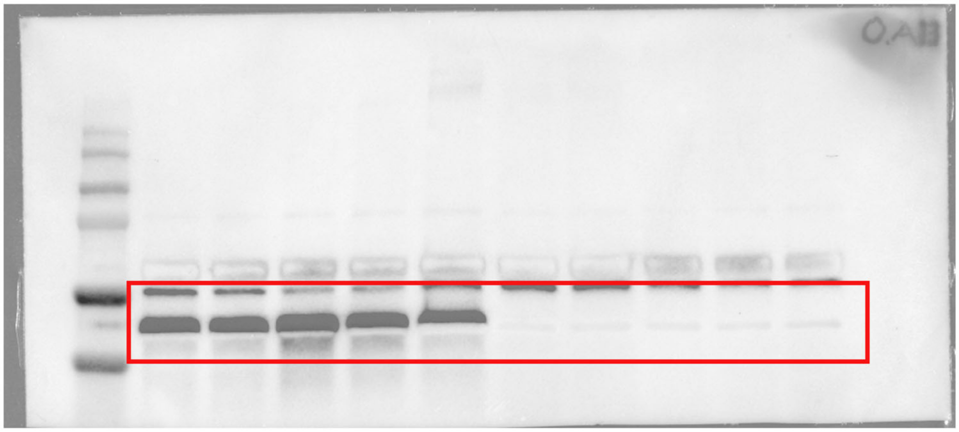

Original western blots

Figure 3F

Beta-actin

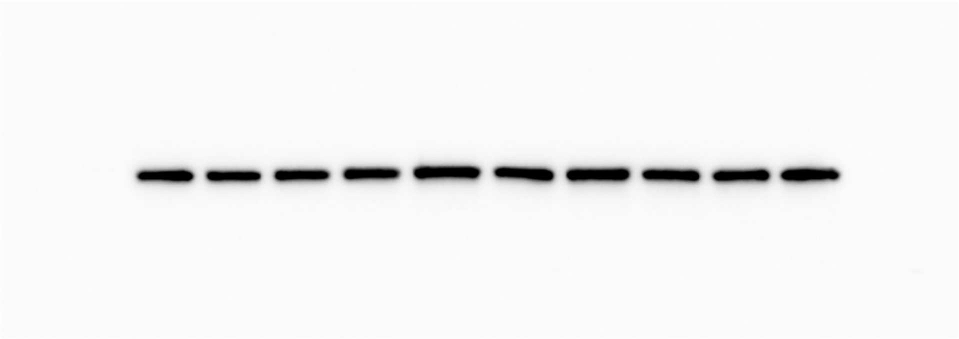

Figure 4A

LC3

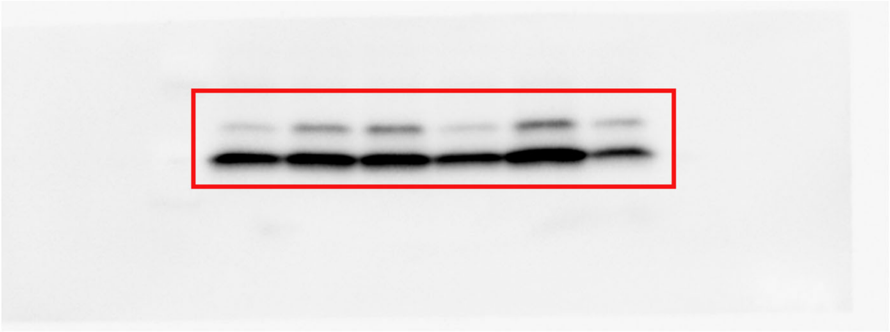

TC-PTP

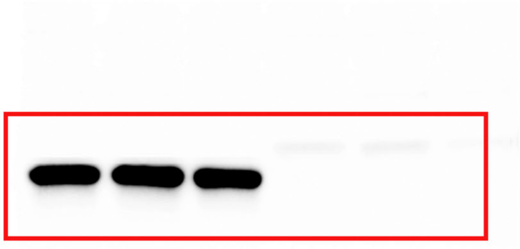

Beta-actin

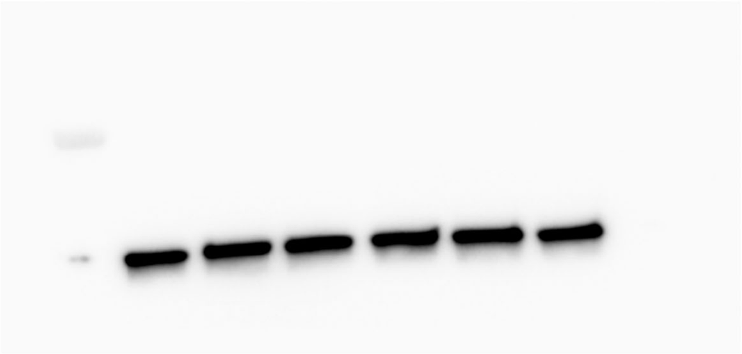

Original western blots

Figure 4C

LC3

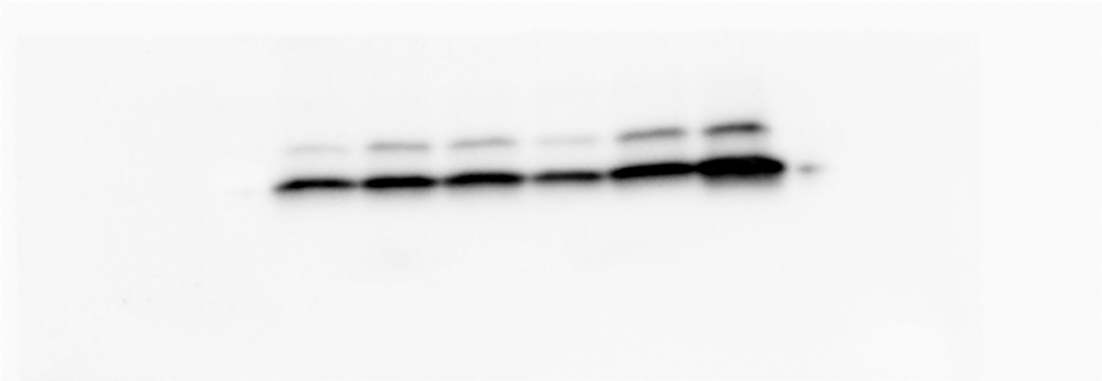

TC-PTP

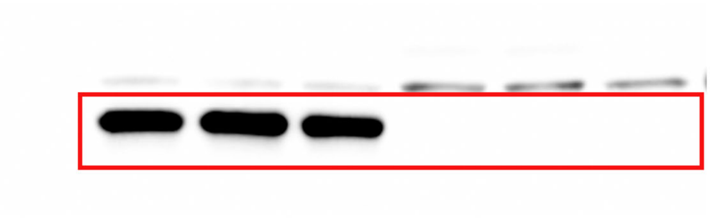

beta-actin

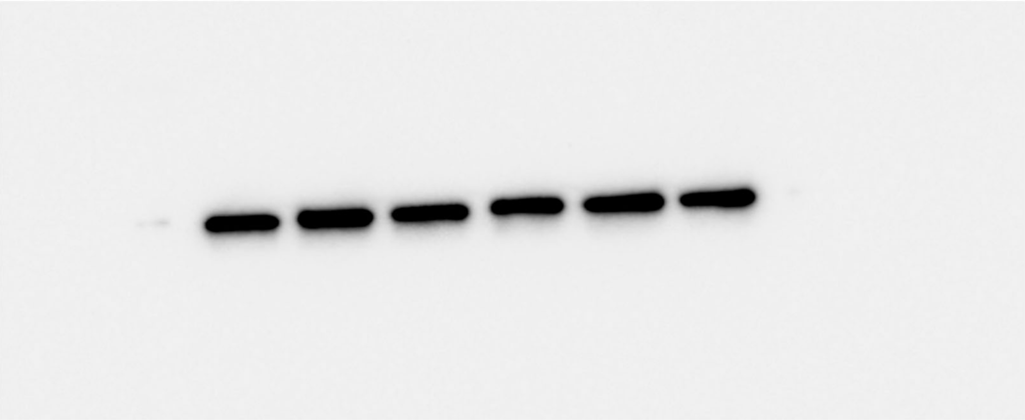

Supplement: Supplementary file 1 — Original data [file 41420_2025_2353_MOESM1_ESM.pdf]
